# National Cancer Institute's Cancer and Aging Priorities

**DOI:** 10.1093/geroni/igab046.1157

**Published:** 2021-12-17

**Authors:** Jennifer Guida

**Affiliations:** National Cancer Institute, Bethesda, Maryland, United States

## Abstract

Modern improvements in cancer detection and treatment coupled with the implementation of population-based cancer prevention and control strategies have contributed to a sustained decline in overall cancer mortality rates. Although this trend is promising, challenges at the nexus of cancer and aging are, in turn, becoming more prominent. Older adults (age 65 years and older) are the largest growing segment of the U.S. population, and aging into older adulthood is disproportionally associated with the incidence of common cancers. Many survivors of childhood cancer will live for decades after cancer treatment and mature into older age. Strategic investments in aging research will contribute to population health by preserving or improving healthspan and ensuring equitable access to – and benefit from – advances in cancer prevention, control, and population science. This presentation will describe ongoing cancer and aging efforts at the National Cancer Institute, including programmatic priorities and current funding opportunities.

